# The Quantity and Quality of Illegally Imported Products of Animal Origin in Personal Consignments into the European Union Seized at Two German Airports between 2010 And 2014

**DOI:** 10.1371/journal.pone.0150023

**Published:** 2016-02-22

**Authors:** Wiebke Jansen, Majella Merkle, Anna Daun, Matthias Flor, Nils Th. Grabowski, Günter Klein

**Affiliations:** 1 Institute of Food Quality and Food Safety, University of Veterinary Medicine Hannover, Foundation, Hannover, Germany; 2 Integrated Veterinary Research Unit, University of Namur, Namur, Belgium; 3 Institute for Veterinary Epidemiology and Biostatistics, Freie Universität Berlin, Berlin, Germany; 4 Federal Institute for Risk Assessment, Berlin, Germany; Linneaus University, SWEDEN

## Abstract

The import of products of animal origin (POAO) in travellers’ personal consignments presents a considerable risk of introducing animal diseases and emerging zoonoses into the European Union. The current regulation (EU) 206/2009 implements strict measures for illegally imported POAO, whereupon non-complying products have to be seized and destroyed regardless. Especially airports serve as global bottlenecks for illegally imported POAO where passenger controls of non-European flights are performed by customs and veterinary services in collaboration. Results of these control measures have to be submitted in the form of annual reports to the European Commission. However, few data on qualities and quantities of seizures have been published so far. In this study, POAO seized at two German airports between 2010 and 2014 were analysed in terms of quantities, qualitative categories and region of origin. In most years considered, more than 20 tonnes POAO were seized at each airport. However, reported amounts of seizures seem to be only the tip of the iceberg as an all-passenger control is not feasible and therefore travellers are only spot-checked. The analysis suggests that the organisational structures of both customs and official veterinary services and their different risk perceptions interfere in completing an effective ban on the illegal import of POAO.

## Introduction

Animal diseases formerly regarded as eradicated such as foot and mouth disease (FMD) as well as classic and African swine fever (CSF/ASF) remain the most serious threats to naive European livestock. Previous outbreaks have been caused by uncommon strains never isolated before in the European Union (EU) [1, 2] while illegal imports of meat and derived products are often supposed to initiate those outbreaks with considerably high socio-economic costs [3, 4].

Food and feedstuff imported by legal trade into the EU are monitored by the official authorities in each member states (MS) and notified via the rapid alert system for food and feed (RASFF; http://ec.europa.eu/food/safety/rasff/index_en.htm). Zoonotic pathogens such as *E*. *coli* and *Salmonella* spp. have been isolated frequently from transcontinentally traded meat [5]. However, illegally imported products of animal origin (POAO) may also harbour zoonotic pathogens which pose an additional risk for public health, especially in case of uncommon strains [6, 7]. The risk of introducing eradicated pathogens is omnipresent as considerable amounts of POAO from endemic countries are continuously introduced into the EU illegally and pose human or animal health risks due to infectious agents. A study carried out at Roissy Charles-de-Gaulle airport (Paris), France in 2008, illegally imported bush meat and fish had been seized from passengers by customs officers and microbiological findings identified unsafe levels of total mesophilic bacteria and zoonotic bacterial pathogens such as *E*.*coli*, *Listeria monocytogenes* and *Staphylococcus aureus* demonstrating the potential public health risks associated with this trade [8, 9].

Little information is available on zoonotic risks stemming from illegally imported POAO and no baseline data on the prevalence of associated zoonoses was presented so far.

All animal diseases and zoonotic outbreaks harm the production of food of animal origin, particularly of essential food proteins and generate hurdles for international trade. These hazards are nowadays increasingly recognised, especially in regard of the fact that non-vaccinating strategies are an essential part of the EU animal health policy, which is aimed at eradicating highly contagious diseases as quickly as possible, and at keeping economic damage to a minimum. The most effective means of doing so is to slaughter and destroy infected or potentially infected animals. For example, with the available testing methods it is impossible to distinguish between an animal that has been vaccinated against FMD and one that is infected. Vaccination therefore makes it impossible to trace infected animals and, consequently, to eradicate animal diseases such as FMD. The slaughter and destruction of infected or potentially infected animals is necessary to maintain the FMD-free status of the EU as a whole. Moreover, the immediate consequence of large-scale vaccination would be that third countries would prohibit the import of all live animals and non-treated products from the EU. This would lead to very severe losses in terms of trade and employment [10]. Eventually, the emergence and worldwide spread of multi-drug resistant organisms and the subsequent lack of therapy options affects all stakeholders. Inappropriate use of therapeutic antimicrobials in human and veterinary medicine, the use of antimicrobials for non-therapeutic purposes as well as the pollution of the environment by antimicrobials is accelerating the emergence of resistance. Increasing global trade and travel favours the spread of antimicrobial resistance between countries and continents [11].

However, POAO may circulate freely between the 28 MS of the EU and three European Free Trade Associations (EFTA) MS Liechtenstein, Norway and Switzerland, here referred to as the common economic area (CEA). Within the CEA, no customs controls or veterinary checks are performed. Hence, the term “import” here relates only to the introduction of POAO from third countries outside the CEA whereas (intra-EU) trade defines the movement of animals and/or POAO within the CEA [12,13].

First legal regulations on personal imports followed the 2001 FMD epidemic in the United Kingdom spreading to continental Europe via the Netherlands [14]. At present, regulation (EU) 206/2009 generally prohibits third country travellers from introducing POAO into the CEA. The ban applies to all POAOs which have not been approved officially for the import into the CEA by specific EU authorized offices in the countries of origin. There are, however, exceptions for the introduction of POAO from certain third countries such as Iceland that present a negligible animal health risk due to their OIE confirmed animal health status, geographical location or trading agreements [15]. Passengers who are not able to present the corresponding certification paper at CEA borders have to pay a fine. The POAO has to be seized and surrendered immediately for official disposal.

In the EU, the border controls are carried out at designated points of entry, so called border inspection posts (BIP) by official veterinarians in collaboration with the customs service. In Germany, uniquely the customs service as a national security agency has the right to retain and control travellers and luggage. On the other hand, the seizure of POAO falls into the competence of the veterinary services guided by their responsible authorities such as the agrarian and/or health and consumers departments of their respective Federal States (German Bundesländer). Since the latter implement EU regulations in this policy field independently from one another, the veterinary services’ role at the BIPs differs from one Federal State to the other.

Approved BIPs are listed in the Commission Decision 2009/821/EC, which is reviewed approximately 3–4 times a year. The Commission Decision (CD) 2014/704/EU contains the latest amendments to the official list. Currently, there are around 300 BIPs in the CEA and 15 BIPs in Germany. Since Germany is placed in central Europe and thus lacks external borders with third countries, German BIPs are located at international airports and seaports. Illegal imports happen likewise for both route as deliberated acts as well as unintended, while seaports have more relevance to the commercial import of foodstuffs, international airports are in particular bottlenecks for the illegal import of animals and POAO by private passengers.

Each EU MS have to report annually to the European Commission which measures they have taken to advertise and enforce the rules on POAO controls. According to Annex VI of regulation (EU) 206/2009, the report has to include the approximate total number of illegal POAO consignments found in personal luggage at CEA entry points during the reporting year as well as the five third countries from which passengers were most regularly found to carry POAO consignments. Furthermore, the approximate amount in kilogrammes of each POAO seized and destroyed from personal luggage ought to be listed and complied in total amounts per category. However, the report does not include the total number of passengers searched p.a. by the customs. On behalf of the European Commission, the Food and Veterinary Office (FVO) verifies to what extent European MS comply with the rules regarding the illegal introduction of POAO and living animals. In Germany, FVO audits were carried out in June and July 2005. It was reported that at one German airport BIP, a total of 3.5 tonnes of meat products and 2.5 tonnes of dairy products were seized between June 2004 and December 2004 [13]. Aggregated amounts for Germany in 2004 were estimated as being approximately 13.3 tonnes of meat products and 17.5 tonnes of dairy products resulting in nearly half the amount seized in 2003 (26 tonnes meat products and 28 tonnes dairy products) [13]. However, existing data appear inconclusive as not only control practices but also documentation procedures vary between MS and single BIPs [16]. A previous study on the illegal import at two German airports estimated for the larger airport an annual amount of approximately 2,800 tonnes of illegally introduced POAO [13, 14]. A study conducted at the Roissy-Charles de Gaulle airport in Paris estimated an illegal import of 3,287 tonnes of meat and fish per year on Air France flights arriving in Paris from Central and West Africa, of which 273 tonnes were supposed to be bush meat [8]. For Great Britain, the illegal import of meat via airfreight passenger luggage, sea freight, and airmail was estimated as being approximately 12,000 tonnes per annum [2]. Apart from these studies, few scientific data were published on the quantities and qualities of illegally imported POAO into the EU market. Our study provides therefore structured data on the amounts, composition and countries of origin of illegally imported POAO seized at two international German airports between 2010 and 2014.

## Material and Methods

This study aims to point out the total amounts, their composition and countries of origin of illegally imported POAO via two international German airports. International Airport 1 (IA 1) is located in Eastern Germany with passenger traffic amounting on average to more than 7 million passengers p.a., mainly via low-cost passenger airlines such as Ryanair and easyJet. Several times a week, and more frequently in the summer months, there are direct flights to third country destinations including Turkey, the Middle East, North Africa, Russia and the Caucasus region. Although there are no direct air routes to Central and South Africa or Asia and the Americas, passengers coming from those regions arrive at IA 1 via other destinations as transit passengers. International Airport 2 (IA 2) is located in Central Germany with passenger traffic amounting on average to almost 57 million passengers p.a., mainly via Star Alliance Airlines such as Lufthansa. Third country connections are served on a daily basis to all world regions all through-out the year.

Figures on passengers between 2010 and 2014 were obtained from the annual reports of the two airports’ operating companies. These figures include inner-CEA flights as well as flights arriving from third countries. The inclusion of inner-CEA flights is methodologically appropriate since figures on CEA passengers include transit passengers from other world regions as well. It is relevant in this regard, that transit passengers who change flights in other CEA countries pass through customs controls only when they enter the CEA but avoid controls when they continue their journey inside the CEA.

Both BIPs conveyed data on POAO seizures including total seizures p.a. of meat products, dairy products and other POAO (mainly honey with combs and products thereof) as well as the estimated weight of each confiscated consignment. The same data is submitted to the European Commission according to Annex VI of regulation (EU) 206/2009. Additionally, IA 1 provided an assessment of the countries of origin from which illegally imported POAO were found most regularly between 2010 and 2014. IA 2 provided the same ranking exclusively for the year 2014. Depending on the seized amounts, the countries were grouped accordingly to major world regions: Asia, the Americas (including North and South America), Central and South Africa, North Africa, Russia and the Caucasus region, as well as Turkey and the Middle Eastern countries. Data were analysed with descriptive statistics and processed in Microsoft ^®^ Excel 2011 and RStudio.

## Results

### Quantities and categories of illegally imported POAO

Over the period investigated the total number of annual passengers at IA 1 ranged from 6.7 million (2013) to 7.3 million (2010, 2014). Total annual seizures ranged from 567 (2013) to 866 (2011) consignments (Table 1), i.e. on average between 1.6 (2013) and 2.4 (2011) seizures per day. The ratio between annual POAO seizures and annual passengers added up to 0.8% (2013, 2014) and 1.2% (2011) respectively. Hence, about one per cent of all passengers passing IA 1 have demonstrably imported POAO illegally. All in all, the mean ratio of weight per seizure increased by 10% between 2010 and 2014 from 3.1 kg/seizure to 3.4 kg/seizure.

**Table 1:** Total passengers, total seizures, seizures/day, seizure-passenger ratio and mean seized amount in kilogrammes per passenger at IA 1 and IA 2, 2010–2014.

	Total passengers		Total seizures		Seizures/day		Seizures/ passengers		Seized amount [kg]/passenger	
Year	IA 1	IA 2	IA 1	IA 2	IA 1	IA 2	IA 1	IA 2	IA 1	IA 2
**2010**	7,297,911	53,013,771	675	6,740	1.85	18.47	0.925%	1.271%	3.07	3.28
**2011**	7,113,989	56,443,657	866	7,015	2.37	19.22	1.217%	1.243%	3.19	3.26
**2012**	7,097,274	57,527,251	796	2,477	2.18	6.79	1.122%	0.431%	3.22	3.38
**2013**	6,727,306	58,042,554	567	721	1.55	1.98	0.843%	0.124%	3.19	3.66
**2014**	7,292,517	59,571,802	611	878	1.67	2.41	0.838%	0.147%	3.43	3.63
**Mean**	**7,105,799**	**56,919,807**	**703**	**3,566**	**1.92**	**9.77**	**1.01%**	**0.64%**	**3.22**	**3.44**

At IA 2, the number of annual passengers increased continuously from 2010 with 53 million up to 59 million passengers in 2014. By contrast, the figures on POAO seizures decreased during the same period. While in 2010, the veterinary service counted 6740 seizures (approximately 18 per day) and in 2011 a total of 7015 seizures (approximately 19 per day), in 2012 this figure decreased about 63% to 2477 seizures (approximately 7 per day). In 2013 a total of 721 and in 2014 a total of 878 consignments (approximately 2 per day, respectively) were seized, indicating a decline of 89% (2013) and 87% (2014) compared to total seizures in the year 2010. Thus, the share of passengers who demonstrably introduced POAO illegally via IA 2 into Germany has decreased significantly from 1.3% in 2010 and 1.2% in 2011 down to 0.4% in 2012 and 0.1% in 2013 and 2014 respectively. The mean ratio of weight per seizure increased slightly but continuously between 2010 and 2014 from 3.3 kg/seizure to 3.6 kg/seizure. Table 1 summarises these figures.

**Fig 1:**
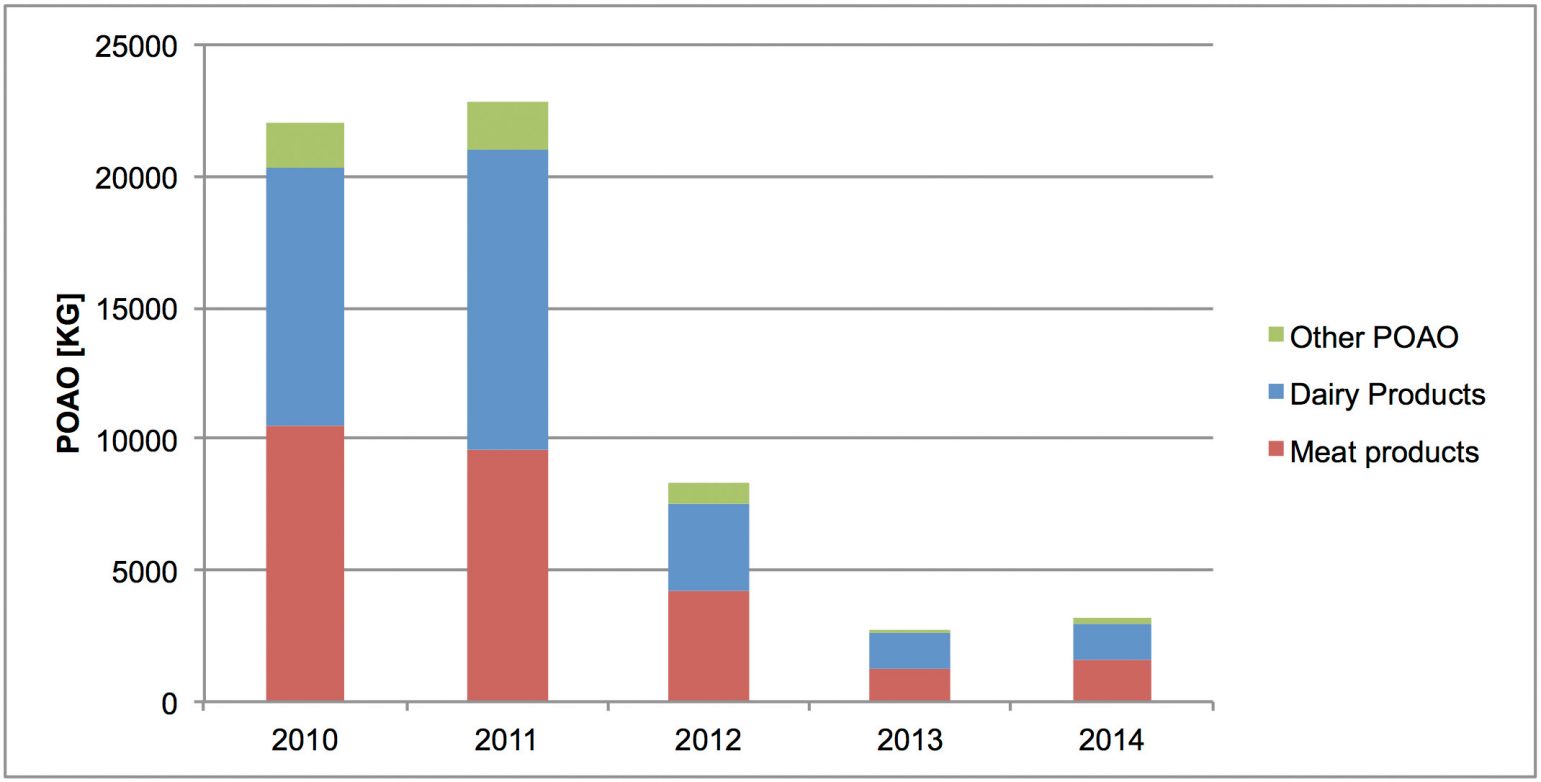
Total amount of seized POAO (in kg) and mean amount of kilogrammes per passenger at IA 1, 2010–2014.

In terms of weight, dairy products were the main POAO category seized at IA 1. In 2010 and 2011, seizures of dairy products reached a total weight of 1.2 tonnes and 1.3 tonnes respectively, whereas meat products were seized to a lesser extent with an amount of 0.8 and 1.2 tonnes during the same years, respectively. In contrast, meat products were seized in larger quantities between 2012 and 2014. The seizure of other POAO (mainly honey with combs, and products thereof) with largest amount recorded in 2011 and 2012 at 0.14 and 0.12 tonnes, respectively (Fig 1).

**Fig 2:**
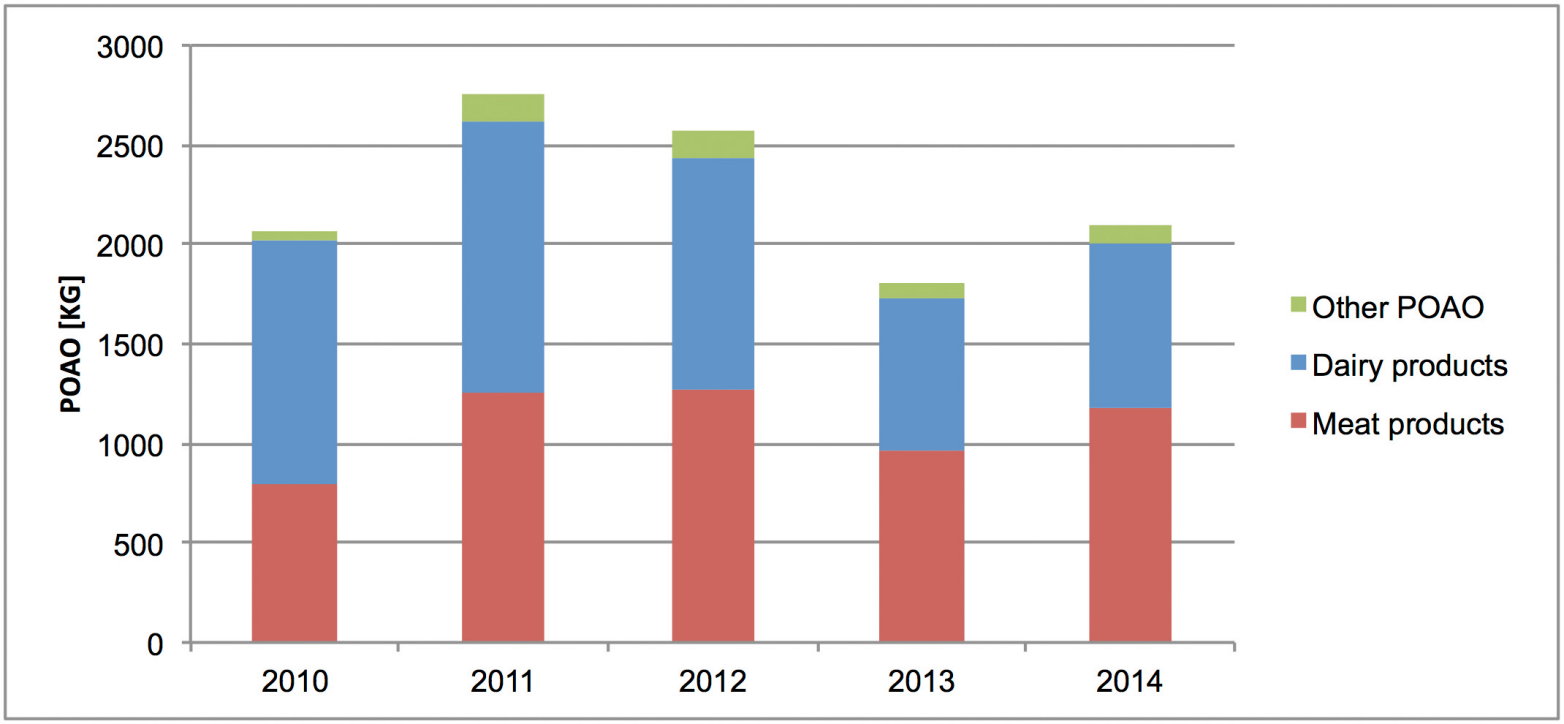
Total amount of seized POAO (in kg) at IA 2, 2010–2014.

At IA 2, regarding the seized quantities of dairy and meat products in 2010 and 2011, dairy products amounted to 9.8 and 11.4 tonnes in 2010 and 2011 whereas 10.5 and 9.5 tonnes of meat products and 1.8 and 1.9 tonnes of other POAO were seized during the same period. In 2012, total seized amounts of POAO were less, including 3.3 tonnes of dairy products (minus 66% compared to 2010), 4.3 tonnes of meat products (minus 60% compared to 2010) and 0.8 tonnes of other seized POAO (minus 55% compared to 2010). As shown in Fig 2, the total amount of seizures decreased even more at IA 2 in the following years: the seized amounts of meat products dropped by 89% in 2013 and 85% in 2014, dairy products by each 86% in 2013 and 2014, and other POAO by 96% in 2013 and 89% in 2014 each compared to the amounts of 2010. However, the ratio of seized products remained similar: In 2013 and 2014, 1.4 tonnes of dairy products, 1.2 and 1.6 tonnes of meat products and 0.06 and 0.2 tonnes of other POAO were seized, respectively. In 2012, intermediate amounts of POAO were seized, including 3.3 tonnes of dairy products, 4.3 tonnes of meat products and 0.8 tonnes of other seized POAO. (Fig 2).

### Origin of illegally imported POAO

**Fig 3:**
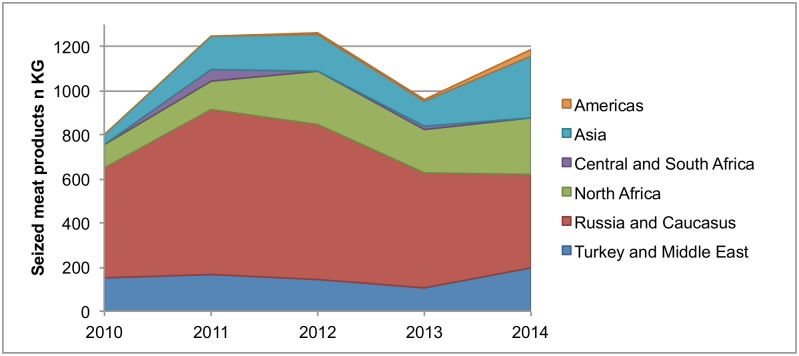
Seized meat products (in kg) at IA 1 according to region of origin, 2010–2014.

At IA 1, a total of 5.5 tonnes of meat products were seized between 2010 and 2014 (Fig 3). More than half of the amount (2.9 tonnes or 53%) derived from travellers arriving from Russia and the Caucasus. Passengers from North Africa (0.9 tonnes or 17%) introduced the second largest share of seized meat products, followed by those arriving Turkey and the Middle East (0.75 tonnes), and via transit flights from Asia (0.73 tonnes), each amounting in 14%. Central and South Africa as well as the Americas (both via transit flights) accounted for 69 kg and 43 kg, or 1%, respectively.

**Fig 4:**
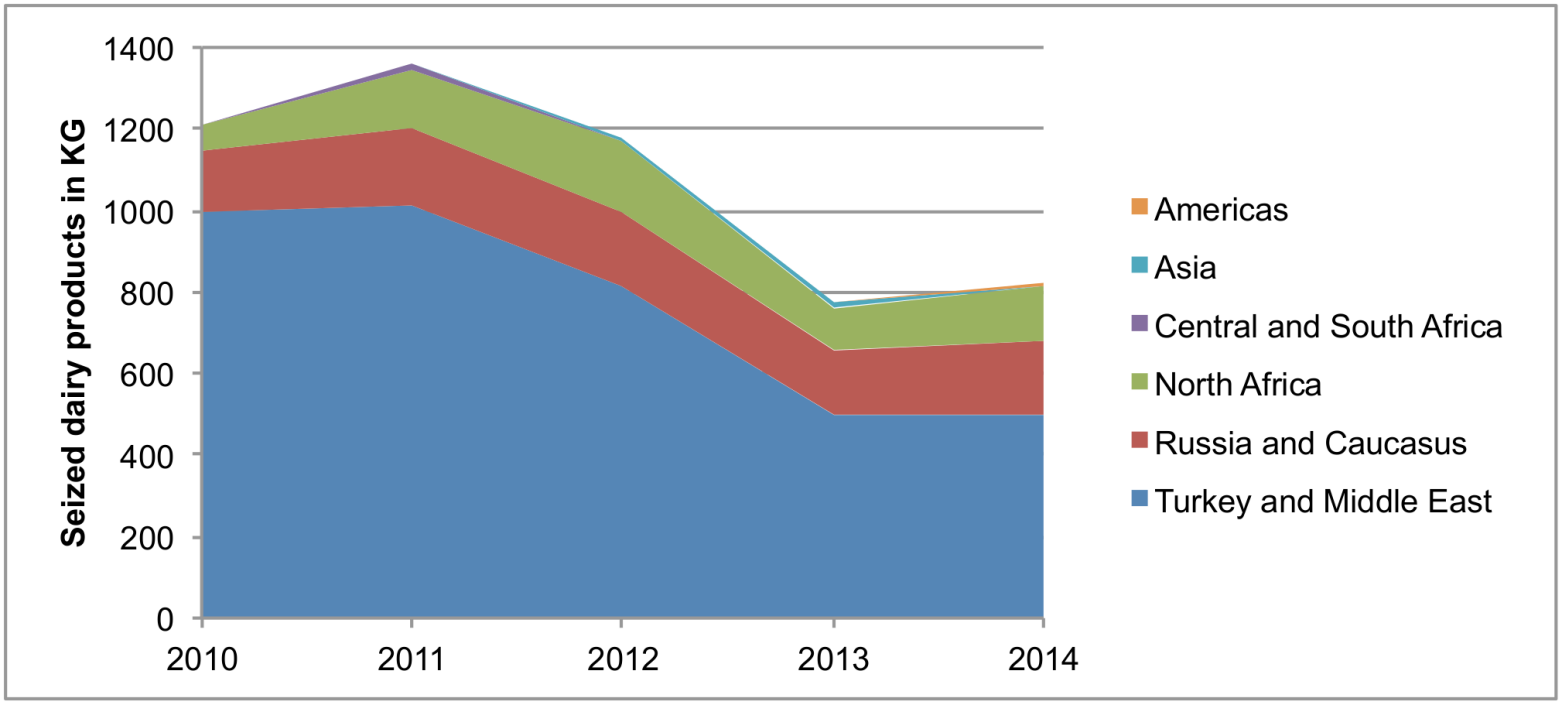
Seized dairy products (in kg) at IA 1 according to region of origin, 2010–2014.

More than two thirds (3.8 tonnes or 71%) of a total of 5.4 tonnes of dairy products seized at IA 1 between 2010 and 2014 derived from travellers arriving from Turkey and the Middle East. Travellers coming from Russia and the Caucasus introduced the second largest quantity (0.83 tonnes or 16%), followed by those from North Africa (0.6 tonnes or 12%). Dairy products from Central and South Africa (28 kg), Asia (20 kg) and the Americas (6 kg) issued in less than 1%, respectively (Fig 4).

Regarding other POAO seized at IA 1 between 2010 and 2014, summarized amounts reach approximately 10% of the recorded meat and dairy POAO amounts. Table 2 shows that the majority (0.3 tonnes or 60%) of the 0.5 tonnes derived from travellers arriving from Turkey and the Middle East, followed by Russia and the Caucasus (0.17 tonnes or 35%), North Africa (200 kg or 3%) and Asia (100 kg or 2%). Other POAO from Central and South Africa accounted for less than 1% with 40 kilogrammes and no seizures were recorded for other POAO deriving from the Americas.

**Table 2:** Other POAO (in kg) seized at IA 1 according to region of origin between 2010–2014.

Year	Turkey and Middle East	Russia and Caucasus	North Africa	Central and South Africa	Asia
**2010**	36	23	0.9	0	0
**2011**	99	33	12.1	0.4	4.1
**2012**	55	62.4	0	0	7
**2013**	53	26	0	0	0
**2014**	57	32	0	0	0
**Sum**	**300**	**176.4**	**13**	**0.4**	**11.1**

At IA 2, 1.6 tonnes of meat products were seized in 2014. Half of this amount was imported by travellers coming from Asia (0.8 tonnes kg or 49%). Turkey and the Middle East accounted for 372 kg (23%), and Russia and the Caucasus for 206 kg (13%). Another share of 110 kg (7%) originated in Central and South Africa, the Americas (91 kg or 6%) and North Africa 28 kg or 2%). Two kilogrammes of meat products were seized from Australia, resulting in less than 1%.

**Fig 5:**
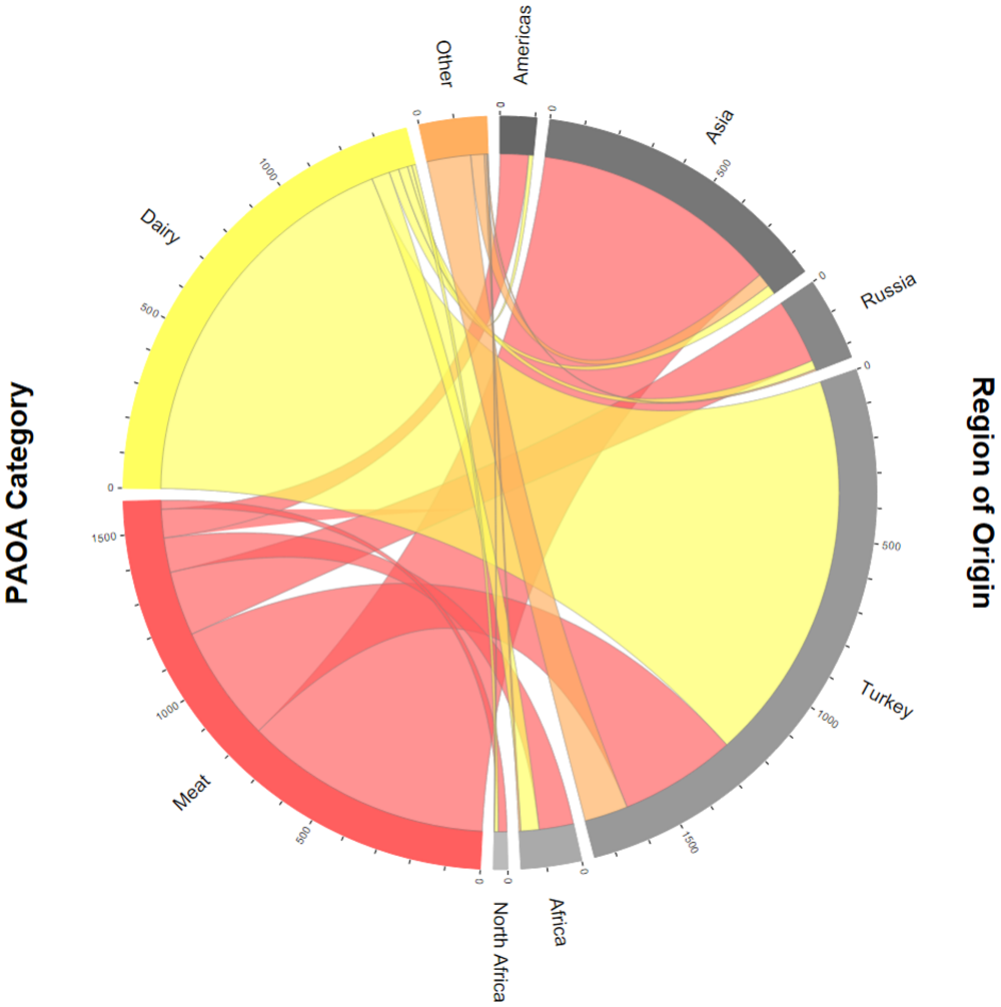
Seized meat products, dairy products, and other POAO in kilogrammes at IA 2 in 2014 according to region of origin. Arc lengths on the outer circle are proportional to the fractions of POAO categories and regions of origin, respectively.

At IA 2 a total of 1.4 tonnes of dairy products were seized, with 90% (1.25 tonnes) deriving from travellers arriving from Turkey and the Middle East. The remaining 10% were brought by passengers coming each from Central and South Africa (58 kg or 4%), Asia (31 kg or 2%), Russia and Caucasus (29 kg or 2%) as well as the Americas (14 kg or 1%) and North Africa (12 kg or 1%). Other POAO were seized to a total of 194 kilogrammes, mainly from Turkey and the Middle East (139 kg or 71%), Asia (43 kg or 22%), Central and South Africa (6 and 5 kg or 3%), and North Africa (1 kg or less than 1%) (Fig 5; results for Australia not shown). An interactive version of Fig 5 is presented as S1 Fig.

## Discussion

Major efforts have been made to eradicate serious animal diseases within the European Union. The current regulation (EU) 206/2009 aims at limiting the introduction of POAO in personal consignments into the CEA [15]. Compiling the results of all EU MS, the total number of illegal consignments of meat and dairy products found in personal luggage at Community entry points was approximately 70,924 in 2009, 81,397 in 2010 and 67,003 in 2011. The amount of meat and dairy products confiscated and/or destroyed from personal luggage as a result of checks implemented at all Community entry points combined was 273 tons in 2009, 212 tons in 2010, and rose to 280 tons in 2011 [17]. Our study shows that in most years we analysed, more than 20 tonnes of POAO were seized at each airport representing almost 10% of the total EU seizures. This highlights the importance of Germany´s international airports as bottlenecks for illegally imported POAO. The average of about 3 kilogrammes POAO per seizure has risen slightly over the observed period. In a study conducted at the Roissy-Charles de Gaulle airport in Paris, the largest seized consignment of bush meat weighted 51 kilogrammes carried by a passenger with no other luggage [6]. 18 illegal African bush meat consignments arising from these seizures at Roissy-Charles de Gaulle airport were analysed microbiological. *E*. *coli* and *Listeria monocytogenes* were each isolated from two samples, *Staphylococcus* spp. were identified in nine of the samples analysed and thereof *S*. *aureus* was detected in four samples. *Salmonella* spp. were not isolated from any sample [9]. A previous study conducted at IA 1 and IA 2 calculated an average weight of 2–2.7 kilogrammes POAO per seizure ranging between more than 20 kilogrammes raw cheese and butter and a single salami sandwich from flight catering [18]. The latter study also assessed zoonotic bacteria in seized POAO at IA 1 and IA 2. Analysing a total of 474 products according to the respective ISO standards, *Salmonella* was found in four meat samples, *Listeria monocytogenes* in nine samples, vero-toxin producing *E*. *coli* in seven samples and *Yersinia enterocolitica* was detected once [18, 19]. In another study, 61,355 passengers were spot-checked at Vienna Airport. The microbiological analysis was performed according to the respective ISO standards and detected *Salmonella* spp. in 1.1% of the samples, *Listeria monocytogenes* in 2.5%, and vero-toxin producing *E*. *coli* in 1.3% of the samples [20, 21]. Yet additional investigation is necessary to estimate baseline data on the prevalence of zoonotic bacteria in illegally imported POAO. However, there are multiple factors that complicate the establishment of those baseline data.

### Control rates

The customs service as a national security agency has the right to retain and control travellers and luggage, and total numbers of passengers searched are not required according to Annex IX of the regulation 206/2009. Therefore, this information is lacking. The number of unknown cases is difficult to estimate, and results have to be interpreted in view of BIPs’ background conditions. Control rates vary over geographical region and time depending on organizational requirements, facilities and coordination between the agencies involved. For instance, veterinarians at IA 1 explained that they rarely miss any relevant flight from third countries because IA 1 is a small airport with only one terminal with one exit that is nearly always being controlled. At IA 1, every passenger is passing the manned custom check after baggage claim before entering the public arrival area. Transit passengers are extremely rare, but they would need in any case to claim their baggage, exit and pass the customs to re-enter the departure area. The customs officers spot check certain exiting passengers and control their identity, hand luggage and further baggage. If violation of EU laws is evident, including the illegal introduction of POAO, the customs officer issues penalties and the passenger has to pay a fine. By contrast, IA 2 has got several terminals with various exits that are frequented by passengers often simultaneously. If transiting through IA 2 to a connecting flight, passengers may be able to have their baggage tagged through to their final destination. Transit passengers will need to exit and declare any items in the hand baggage that are liable for duty. The passengers may choose according to their status two exit aisles. One is indicated for passengers who have “Nothing to declare” and another for passengers who need to pass the customs check to declare goods. Nonetheless, customs officers perform additionally spot-checks and retrain travellers leaving via the “Nothing to declare” exit to control their identity, hand luggage and further baggage. If customs regulations are violated, the officer issues penalties and the passenger has to pay a fine. Other legal areas as Reg. (EU) 206/2009 or the Convention on International Trade in Endangered Species of Wild Fauna and Flora (CITES), are not the scope and area of the customs responsibility. Therefore, veterinary controls were implemented at IA 2 continuously full-time for seven days a week. Comparing figures with IA 1, 1.3% and 1.2% of the travellers were found to import POAO illegally in their luggage in the years 2010 and 2011. Due to changes in the human resource management, the control frequencies at IA 2 were changed in summer 2012 from seven days a week full-time (approximately 49 hours a week) to five days a week part-time (approximately 24 hours a week) in the following years. In 2014, a total of 0.1% of the travellers was found to be carrying illegal POAO in their personal consignments. This shows a disproportional decline of more than 90% compared to 2010, where 1.3% travellers were found to be carrying illegal POAO. These differences might explain why in 2013 and 2014 the amounts of seized POAO at both airports were on a rather similar level although total passenger numbers at IA 2 were about eight times higher than at IA 1.

The infrastructure, development and operating airlines differ significantly between the two airports. IA 2 with approximately 57 million passenger p.a., serves eight times more passengers than IA 1. The seized amounts at IA 2 in the years 2013 and 2014 dropped due to the reduced control time-frames disproportionally by more than 90% and not as expected about 50%, ultimately resulting in fewer seizures than at the much smaller IA 2. As neither the destination, the number of connections nor the airlines changed in these years significantly, the drop seems to be due to the changed control time-frames. Though, the controls seem to have become more efficient as the average amount of POAO per seizure has risen over the years. The fact that seizures at IA 2 were much higher in previous years (2010, 2011) was explained with reference to a higher threat perception in the years following the 2005 avian flu. In April 2005, an outbreak of a high pathogenic avian influenza (HPAI) causing mass die-offs in wild birds was reported at Qinghai Lake in central China. Overall, more than 6000 congregating migratory birds from different species died until June and human cases were reported to end fatal. The associated H5N1 virus was spread among migratory geese and it was suggested that the virus may be carried along winter migratory routes [22]. These first die-offs and the following cases spreading from Eastern Asia to Europe during the year 2005 were alarming stakeholders worldwide. Subsequently, the veterinary personnel’s working hours at IA 2 were exceptionally flexible during these years, covering flights both early in the morning and late at night. However, without facing imminent threats, the veterinary service working hours have been reduced.

### Interagency coordination

In the absence of elaborate rules that would define in what way customs and veterinary services coordinate their work, different local arrangements have been implemented. Customs’ role ranges from stopping the passengers while the seizure is effectively carried out by the veterinary service up to realizing the seizure themselves while the veterinary service is only called afterwards [16]. At IA 1, the veterinary service is usually not present at the customs check. Every time POAO is found in any luggage the customs takes over for seizure. In contrast, no mutually shared assistance is implemented at IA 2. As at IA 2 multiple flights arrive continuously, passengers are often served simultaneously at different terminals and even more exits. As a result, the veterinary service’s presence at the customs check is reduced significantly. The customs and official veterinarian´s competences and area of responsibilities are strictly separated in this case. Thus, customs might call the veterinary service after having seized any POAO consignment or deliver it to the veterinarians afterwards. However, since POAO seizure is not part of customs’ organisational objectives, cooperation is rather driven by good will or individual favour than by mandate or structural incentives. Nonetheless, even if customs would focus on seizing illegal POAO, they are neither supposed to issue the administrative orders and penalties, nor to surrender the seized POAO for official disposal. This is however no exception, customs at several German airports are not able to surrender the seized POAO for official disposal as no facilities for hazardous waste are provided inside the terminals [16]. To foster interagency collaboration, a waste management system was implemented inside the terminals at IA 2 in 2010 and 2011. However, the surveillance of the hazardous waste has been a major effort. Only daily disposal on behalf of the veterinary authorities remained possible as otherwise the tracing and obligatory biosecurity measures applying to the seized POAO could not be guaranteed. This led to significant costs, yet the effort was disproportional when working times were altered in 2010 and a system was implemented as described above.

### Organisational objectives and risk perceptions

Since complete passengers controls are only feasible in exceptional circumstances, the success of spot-check controls depends on appropriate indicators for the targeted goods. As a consequence of different organizational objectives and, as a consequence thereof, risk perceptions of customs vs. veterinary agencies focus their attention on different types of passengers and luggage. In terms of organisational guidelines, customs focus primarily on hazards such as weapons, drugs, pirated products as well as cash and forged money. From customs’ point of view, POAO are of marginal interest as those violations are neither their initial scope nor area of responsibility. By contrast, for the veterinary service, which is to collaborate with customs in this matter, the illegal import of POAO and animals is the occupational focus of attention. The veterinary services are mandated by the responsible authorities at the level of the Federal States. The latter assess risks according to the prevalence of animal diseases in third countries. Their assessments are based on data from multiple international organisations including the Emergency Prevention System (EMPRES; http://www.fao.org/ag/againfo/programmes/en/empres/home.asp) by the FAO (http://www.fao.org/home/en/), the European Animal Disease Notification System (ADNS; http://ec.europa.eu/food/animal/diseases/adns/index_en.htm) by the European Commission (http://ec.europa.eu/index_en.htm), Eurosurveillance (http://www.eurosurveillance.org/) by the European Center for Disease Prevention Control (ECDC; http://ecdc.europa.eu/en/Pages/home.aspx), ProMedMail (http://www.promedmail.org/), the Rapid Alert System for Food and Feed (RASFF) by the European Commission and the World Animal Health Information Database (WAHID; http://www.oie.int/wahis_2/public/wahid.php/Wahidhome/Home) by the OIE (http://www.oie.int/). Further statistics are drawn from the BIPs own documentation on seizures according to (EU) regulation 206/2009 (Annex VI). On the basis of these data a priority list of risky flights is created which is to guide the veterinary service’s focus of attention. The very dissenting approaches and risk perceptions of the customs and the veterinarians do not prioritise the same countries, neither the same airlines nor an aligned selection of controlled passengers. Moreover, the customs services only occasionally seize POAO even if they might be found in the luggage of the controlled passengers. The conflict of interests and disproportionate success rates consequently lead to major discord in responsibilities and collaboration as well as unpredictable cooperation.

### Travellers’ risk perception

Although information on the POAO import ban is conveyed to the public by MS authorities, travellers’ risk awareness is marginal: according to expert interviews at both airports considered in this study, most POAO are imported by expatriates on their flight back from their countries of origin. The qualitative analysis of seized POAO conducted in this study shows that imports are typically handmade and traditional and local foodstuffs reflect culturally enrooted consumption patterns [23]. Accordingly, the vast majority of seized dairy products was imported from Turkey (71%, IA 2) and the Middle East (90%, IA 1), most meat products derived from Asia (49%, IA 2) as well as Russia and Caucasus (53%, IA 1), and the bulk of other POAO, in particular honey with combs, derived from Turkey (60%, IA 2) and the Middle East (71%, IA 2), reflecting common food consumption patterns [23].

Focus group discussions with travellers who have imported POAO indicate that most participants were aware of the general POAO ban for the CEA but did not understand the reasons behind. They were convinced that the food they carry was of high quality and were not able to connect it to any serious risk. These cognitions are accompanied with selective perception patterns. For example, a wide-spread EU poster which explains the prohibition on POAO imports was only partly understood: The crossed out symbols for milk, ham and cheese were well-known among the participants, but the bold letters “Keep animal diseases out of the European Union” were generally overlooked [24]. Furthermore, also the awareness of food safety hazards needs to be heightened, as even contaminations with “harmless” bacteria like *E*. *coli* can be of concern [5, 7, 25].

## Conclusions

Globalisation, international trade and the ever-growing flow of goods and people enable pathogens to travel worldwide. International airports serve as bottlenecks for the illegal import of POAO potentially containing animal diseases and zoonotic pathogens as travellers continue to introduce illegally POAO from non-EU countries. First attempts were made to characterise the quantity and quality of illegally introduced POAO and their pathogenic potential, but further analyses are essential. Border inspection requires well-organised cooperation between all involved services to limit the illegal import of POAO efficiently. Mutually shared information and competences on a European level of customs and official veterinary services would improve and foster performance, trust, as well as rights and duties. The supply, surveillance and financing of POAO disposal needs to be re-organised and should include the airports as well as the international passenger transport operators. Hand luggage checks should include in future as well the control for POAOs. Better equipment such as for example scanning gear, detector dogs and most important, more human resources are crucial to identify and confiscate illegal imports of POAO from entering the CEA. Nonetheless, there is an urgent need to increase awareness to ensure that travellers do not inadvertently contravene regulations. Tailored communication campaigns and information on the EU regulations already at the departure in the native languages that also address expatriates might foster the awareness towards serious animal diseases that have been eradicated in the CEA.

## Supporting Information

S1 Fig Interactive version (chorddiagram) of Fig 5 with seized meat products, dairy products, and other POAO in kilogrammes at IA 2 in 2014 according to region of origin. Arc lengths on the outer circle are proportional to the fractions of POAO categories and regions of origin, respectively.
(HTML)
